# Oral Vaccination of Mice With *Trichinella spiralis* Putative Serine Protease and Murine Interleukin-4 DNA Delivered by Invasive *Lactiplantibacillus plantarum* Elicits Protective Immunity

**DOI:** 10.3389/fmicb.2022.859243

**Published:** 2022-05-03

**Authors:** Ying Xue, Bo Zhang, Nan Wang, Hai-Bin Huang, Yu Quan, Hui-Nan Lu, Zhi-Yu Zhu, Jun-Yi Li, Tian-Xu Pan, Yue Tang, Yan-Long Jiang, Chun-Wei Shi, Gui-Lian Yang, Chun-Feng Wang

**Affiliations:** ^1^College of Veterinary Medicine, Jilin Agricultural University, Changchun, China; ^2^Jilin Provincial Engineering Research Center of Animal Probiotics, Jilin Provincial Key Laboratory of Animal Microecology and Healthy Breeding, Jilin Agricultural University, Changchun, China; ^3^Key Laboratory of Animal Production and Product Quality Safety of Ministry of Education, Jilin Agricultural University, Changchun, China

**Keywords:** *Trichinella spiralis*, invasive *Lactobacillus plantarum*, Ts-ADpsp, murine IL-4, DNA vaccine

## Abstract

Trichinellosis is a serious zoonotic parasitic disease caused by *Trichinella spiralis* (*T. spiralis*) that causes considerable economic losses for the global pig breeding and food industries. As such, there is an urgent need for a vaccine that can prevent *T. spiralis* infection. Previous studies have reported that recombinant invasive *Lactococcus lactis* (LL) expressing *Staphylococcus aureus* fibronectin binding protein A (LL-FnBPA+) can transfer DNA vaccines directly to dendritic cells (DCs) across an epithelial cell monolayer, leading to significantly higher amounts of heterologous protein expression compared to non-invasive *Lactococcus lactis*. In this study, the invasive bacterium *Lactiplantibacillus plantarum* (*L. plantarum*) expressing FnBPA was used as a carrier to deliver a novel oral DNA vaccine consisting of *T. spiralis* adult putative serine protease (Ts-ADpsp) and murine interleukin (IL)-4 DNA to mouse intestinal epithelial cells. Experimental mice were orally immunized 3 times at 10-day intervals. At 10 days after the last vaccination, mice were challenged with 350 *T. spiralis* infective larvae by oral inoculation. Immunization with invasive *L. plantarum* harboring pValac-Ts-ADpsp/pSIP409-FnBPA induced the production of anti-Ts-ADpsp-specific IgG of serum, type 1 and 2 helper T cell cytokines of mesenteric lymph node (MLN) and spleen, secreted (s) IgA of intestinal lavage, and decreased *T. spiralis* burden and intestinal damage compared to immunization with non-invasive *L. plantarum* expressing Ts-ADpsp (pValac-Ts-ADpsp/pSIP409). Thus, invasive *L. plantarum* expressing FnBPA and IL-4 stimulates both mucosal and cellular immune response to protect against *T. spiralis* infection, highlighting its therapeutic potential as an effective DNA vaccine for trichinellosis.

## Introduction

Trichinellosis is a foodborne zoonosis caused by *Trichinella spiralis* (*T. spiralis*) (Dupouy-Camet, 2009). Humans and animals are infected with *T. spiralis* mainly through the consumption of raw or undercooked meat contaminated by muscle larvae (ML) (Pozio, 2007). *T. spiralis* infection not only causes considerable economic losses for the pig breeding industry but also threatens human health. *T. spiralis* infections have been reported in 55 countries, and China is a major endemic area of trichinellosis (Cui et al., 2007). As such, there is a need for a safe and effective vaccine that can prevent *T. spiralis* infection in domestic animals and humans.

Proteases hydrolyze peptide bonds are classified into 4 types according to the active site residue or catalytic mechanism, namely, serine, cysteine, aspartic proteases, and metalloproteases (Donaldson et al., 1993). The serine protease plays an important role in the process of parasite infection (Gao et al., 2018). The excretory/secretory products and crude extracts of *T. spiralis* contain serine proteases that can hydrolyze host structural proteins and serve as mechanical and humoral barriers during infection (Ros-Moreno et al., 2000). Serine proteases are also involved in blood clotting, reproduction, and evasion of host immune surveillance (Todorova, 2000; Dzik, 2006; Balasubramanian et al., 2010). Putative serine proteases are distributed in the inner epidermis and esophagus of *T. spiralis* and participate in molting and digestion (Trap et al., 2006). Previous studies have reported that the recombinant *T. spiralis* putative serine protease was sensitive and specific for the measurement of anti-Trichinella IgG, and could act as a potential early diagnostic antigen of trichinellosis (Sun et al., 2018). Thus, we selected *T. spiralis* adult putative serine protease (Ts-ADpsp) protein as an oral DNA vaccine antigen to evaluate its immunogenicity and efficacy against *T. spiralis* infection in mice.

Cytokines are important regulators of the immune response and used as vaccine adjuvants. Interleukin (IL)-4 was shown to promote the excretion of intestinal worms in *T. spiralis*-infected mice, possibly through interaction with IL-13 *via* IL-4 receptor; this resulted in the activation of signal transducer and activator of transcription (STAT)6, stimulation of T and B cell differentiation and proliferation, mast cell degranulation, intestinal mucus secretion, and increased intestinal motility (Knight et al., 2000; Urban et al., 2000; Finkelman et al., 2004).

Some of the *lactic acid bacteria* (*LAB*) are non-pathogenic probiotics that have beneficial effects against parasite infections (Travers et al., 2011). Oral inoculation with the probiotic *Lactiplantibacillus casei* markedly reduced adult worm and muscle larvae burden in *T. spiralis*-infected mice (Bautista-Garfias et al., 2001). *L. plantarum* is a LAB that tolerates acid and bile salts and can adhere to intestinal epithelial cells, allowing it to permanently colonize the intestine (Kato et al., 1999). *L. plantarum* NC8 isolated from silage is widely used as a host bacterium for expressing foreign proteins (Anbazhagan et al., 2013). *Staphylococcus aureus* invades host cells through fibronectin-binding proteins (FnBPA and FnBPB) expressed on the bacterial cell surface that bind α5β1 integrin on the host cell membrane (Liu et al., 2018). Most of the delivery vehicle bacteria are degraded by the phagolysosome and then the delivered plasmid DNA is released and transcribed through the host cell nuclear transcription system (Azevedo et al., 2015). Expression of FnBPA in recombinant *Lactococcus lactis* significantly improves the efficiency of mammalian cell invasion, with the target gene delivered into the host cell (Innocentin et al., 2009). Invasive *L. plantarum* expressing the FnBPA protein reduced cecal damage and decreased anticoccidial index while increasing the rate of relative weight gain in chickens infected with *Eimeria tenella* (Zhang et al., 2020). These findings highlight the potential for using invasive *L. plantarum* as a carrier for DNA vaccines.

In the present study, we developed a DNA vaccine coexpressing Ts-ADpsp and mouse (m)IL-4 delivered by invasive *L. plantarum* and evaluated its protective efficacy against *T. spiralis* infection in mice. Vaccinated mice produced high levels of anti-Ts-ADpsp-specific antibody and showed strong cellular and mucosal immune responses, indicating the induction of protective immunity against trichinellosis.

## Results

### Construction and Expression Analysis of Recombinant Plasmids

**FIGURE 1:**
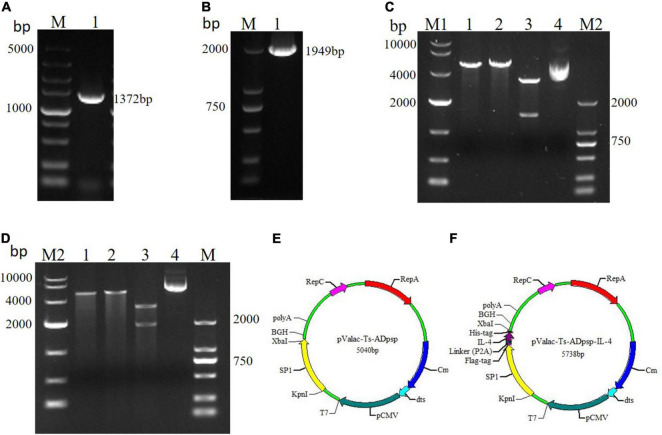
Construction of recombinant plasmids pValac-Ts-ADpsp and pValac-Ts-ADpsp-IL-4. **(A)** Expression of the Ts-ADpsp gene in the tongue muscles of mice challenged with *T. spiralis* muscle larvae. **(B)** PCR amplification of the Ts-ADpsp-IL-4 fragment. **(C)** Identification of the Ts-ADpsp insert in plasmids extracted from randomly picked pValac-Ts-ADpsp colonies after Cm selection. Lane 1: plasmids digested with *Kpn*I; lane 2: plasmids digested with *Xba*I; lane 3: plasmids digested with *Kpn*I/*Xba*I; lane 4: pValac-Ts-ADpsp plasmid. **(D)** Identification of the Ts-ADpsp-IL-4 insert in plasmids extracted from randomly picked pValac-Ts-ADpsp-IL-4 colonies after Cm selection. Lane 1: plasmids digested with *Kpn*I; lane 2: plasmids digested with *Xba*I; lane 3: plasmids digested with *Kpn*I/*Xba*I; lane 4: pValac-Ts-ADpsp-IL-4 plasmid. Genes encoding Ts-ADpsp and Ts-ADpsp-IL-4 were inserted into the pValac vector, producing pValac-Ts-ADpsp **(E)** and pValac-Ts-ADpsp-IL-4 **(F)**, respectively.

The amplified Ts-ADpsp and Ts-ADpsp-IL-4 gene fragments were approximately 1,372 and 1,949 bp, respectively (Figures 1A,B). The Ts-ADpsp and Ts-ADpsp-IL-4 cDNA sequences were separately ligated into the eukaryotic expression vector pValac, and the ligated samples were transformed into *E. coli* TG1 competent cells. Digestion with *Kpn*I/*Xba*I restriction enzymes showed that recombinant plasmids extracted from randomly picked pValac-Ts-ADpsp and pValac-Ts-ADpsp-IL-4 colonies cultured in Luria–Bertani (LB) solid medium with chloramphenicol (Cm) contained an insert of about 1,372 and 1,949 bp, respectively (Figures 1C,D). The presence of pValac-Ts-ADpsp and pValac-Ts-ADpsp-IL-4 in the clones was confirmed by gene sequencing (Figures 1E,F).

**FIGURE 2:**
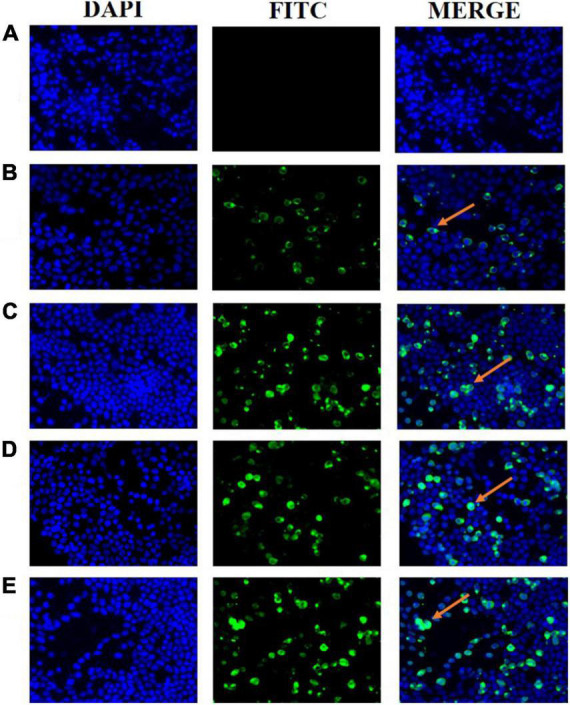
Expression of recombinant plasmids pValac-Ts-ADpsp and pValac-Ts-ADpsp-IL4 *in vitro* by immunofluorescence analysis. Non-transfected cells acted as the negative control **(A)** and pValac with GFP-expressing transfected cells served as the positive control **(B)**. Ts-ADpsp expression in HEK-293T cells transfected with pValac-Ts-ADpsp was observed by IFA using anti-Flag tag monoclonal antibody, FITC-conjugated goat anti-mouse IgG and DAPI **(C)**. Ts-ADpsp-IL-4 expression in HEK-293T cells transfected with pValac-Ts-ADpsp-IL-4 was observed by IFA using anti-Flag tag monoclonal antibody, FITC-conjugated goat anti-mouse IgG and DAPI **(D)**. Ts-ADpsp-IL-4 expression in HEK-293T cells transfected with pValac-Ts-ADpsp-IL-4 was observed by IFA using anti-His tag monoclonal antibody, FITC-conjugated goat anti-mouse IgG and DAPI **(E)**. Arrows indicate the expression of the target protein in HEK-293T cells.

**FIGURE 3:**
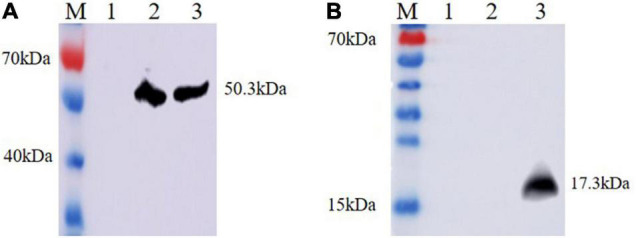
Analysis of Ts-ADpsp and Ts-ADpsp-IL-4 proteins expression by western blotting. Ts-ADpsp and Ts-ADpsp-IL-4 proteins were detected by western blotting using anti-Flag **(A)** and anti-His **(B)** monoclonal antibodies as primary antibodies and HRP-conjugated goat anti-mouse IgG as the secondary antibody. M: protein molecular weight marker; lane 1: pValac/pSIP409 plasmid; lanes 2: pValac-Ts-ADpsp/pSIP409 plasmid and 3: pValac-Ts-ADpsp-IL4/pSIP409-FnBPA plasmid expressed in HEK-293T cells, respectively.

Ts-ADpsp and IL-4 gene fragments were inserted into Flag tag and His tag, respectively. (HEK)-293T cells transfected with pValac-Ts-ADpsp plasmid of human embryonic kidney were incubated with anti-Flag monoclonal antibody (Figure 2C), and 293T cells transfected with pValac-Ts-ADpsp-IL-4 plasmid were incubated with anti-Flag monoclonal antibody (Figure 2D) or incubated with anti-His monoclonal antibody (Figure 2E), and then all the samples were incubated with FITC-conjugated goat anti-mouse IgG, respectively. pValac with GFP-expressing transfected cells was the positive control (Figure 2B) and non-transfected cells were the negative control (Figure 2A). Finally, the samples were treated with DAPI and fluorescence was observed by confocal microscopy. Intense immunoreactivity was observed in cells expressing the Ts-ADpsp and Ts-ADpsp-IL-4 fusion proteins but not in non-transfected cells, suggesting that ADpsp and Ts-ADpsp-IL-4 fusion proteins were successfully expressed in 293T cells. Western blotting revealed 2 protein bands of 50.3 and 17.3 kDa, which are consistent with the sizes of the native proteins (Figures 3A,B). On western blotting analysis, two specific bands of 50.3 and 17.3 kDa were exhibited, and the molecular size of the Ts-ADpsp and IL-4 proteins was consistent with the predicted sizes (Figures 3A,B). The results suggested that two proteins Ts-ADpsp and IL-4 linked by P2A could be sheared when translated in HEK-293T cells and these proteins were expressed independently.

### *Lactiplantibacillus plantarum* Invasion of BHK-21 Cells

**FIGURE 4:**
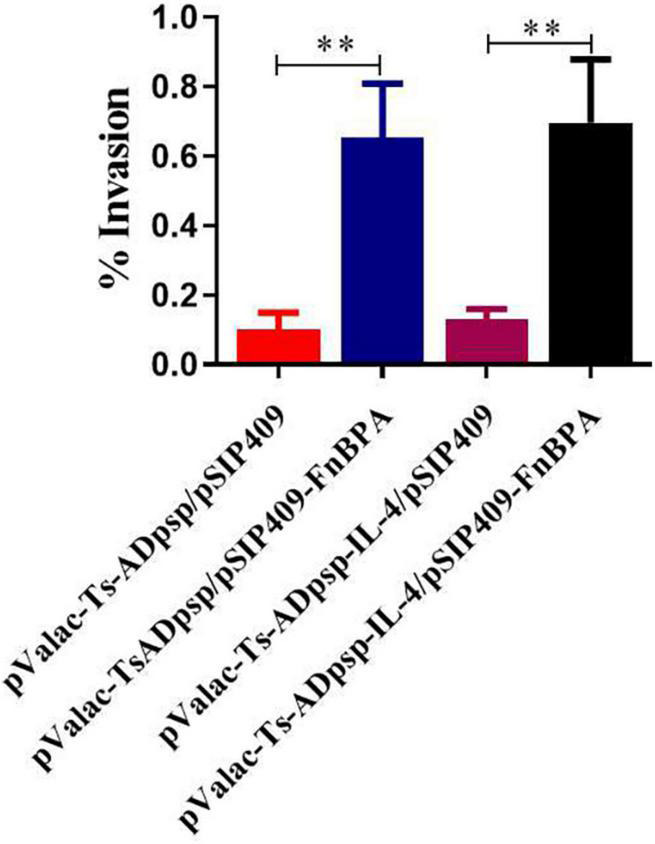
Invasion rates of invasive and non-invasive recombinant *L. plantarum* in BHK-21 cells. Recombinant *L. plantarum* was cocultured with BHK-21 cells and cultured in solid MRS (Em, Cm) at 37°C for 24 h; the number of intracellular bacteria was counted to calculate cell invasion rates. All experiments were independently repeated at least 3 times. Data are shown as means ± standard deviations; *n* = 3. **P < 0.01.

To assess the invasive capacity of invasive *L. plantarum in vitro*, recombinant *L. plantarum* cells were cocultured with BHK-21 cells and the number of intracellular bacteria was counted after 24 h. The invasion rate was higher with invasive *L. plantarum* than with non-invasive *L. plantarum* (*p* < 0.01; Figure 4), suggesting that FnBPA enhanced the ability of *L. plantarum* to invade host cells.

### Analysis of Humoral Immunity

**FIGURE 5:**
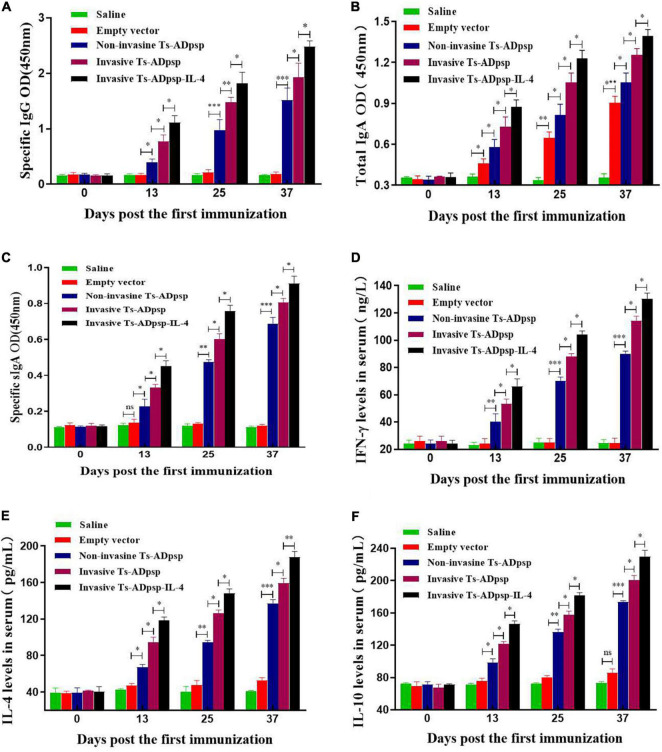
Immune profiles following *T. spiralis* infection with or without vaccination. Levels of anti-Ts-ADpsp-specific IgG **(A)**, total IgA **(B)**, anti-Ts-ADpsp-specific IgA **(C)**, IFN-γ **(D)**, IL-4 **(E)**, and IL-10 **(F)** in sera or intestinal lavage of mice orally administered saline, empty vector, pValac-Ts-ADpsp/pSIP409, pValac-Ts-ADpsp/pSIP409-FnBPA, or pValac-TsADpsp-IL-4/pSIP409-FnBPA 10 days after each immunization were detected by ELISA. Data are presented as mean ± SEM (*n* = 5 mice per group). *P < 0.05, **P < 0.01, ***P < 0.001.

To detect mucosal and humoral immune responses elicited by recombinant *L. plantarum*, serum and intestinal lavage collected from mice were analyzed by indirect ELISA. Mice immunized with recombinant *L. plantarum* pValac-Ts-ADpsp/pSIP409, pValac-Ts-ADpsp/pSIP409-FnBPA, and pValac-Ts-ADpsp-IL-4/pSIP409-FnBPA had a higher titer of anti-Ts-ADpsp-specific IgG than the control groups (empty vector and saline) (*P* < 0.05), and the titer increased with each boost immunization. Notably, mice immunized with pValac-Ts-ADpsp-IL-4/pSIP409-FnBPA had a higher specific IgG antibody level than the other four groups at 10 days after each immunization, with a peak at 10 days after the last immunization (Figure 5A).

Mice immunized with pValac-Ts-ADpsp/pSIP409, pValac-Ts-ADpsp/pSIP409-FnBPA, and pValac-Ts-ADpsp-IL-4/pSIP409-FnBPA had higher total sIgA than control groups after each immunization (*P* < 0.05). Total sIgA level was higher in the empty vector group than in the saline group (*P* < 0.05), implying that sIgA production was stimulated by *L. plantarum* (Figure 5B). Mice in the invasive *L. plantarum* Ts-ADpsp-IL-4 group produced a larger amount of specific sIgA compared to those in the invasive (*P* < 0.05) and non-invasive (*P* < 0.05) *L. plantarum* Ts-ADpsp groups (Figure 5C). Statistically significant differences were observed between vaccinated mice and control mice not only in total IgA (*P* < 0.05, < 0.01, and < 0.001), but also in specific IgA (*P* < 0.05, < 0.01, and < 0.001) (Figures 5B,C).

Administration of recombinant *L. plantarum* pValac-Ts-ADpsp/pSIP409, pValac-Ts-ADpsp/pSIP409-FnBPA, and pValac-Ts-ADpsp-IL-4/pSIP409-FnBPA induced high levels of IFN-γ (*P* < 0.01 and < 0.001), IL-4 (*P* < 0.05, < 0.01, and < 0.001), and IL-10 (*P* < 0.05, < 0.01, and < 0.001) relative to control groups, specifically in the invasive *L. plantarum* Ts-ADpsp-IL-4 group 10 days after each immunization (Figures 5D–F). These data suggest that *L. plantarum* expressing Ts-ADpsp antigen induced the type 1 and 2 T helper cell (Th1 and Th2, respectively) cellular immune responses.

### Analysis of Cytokine Profile

**FIGURE 6:**
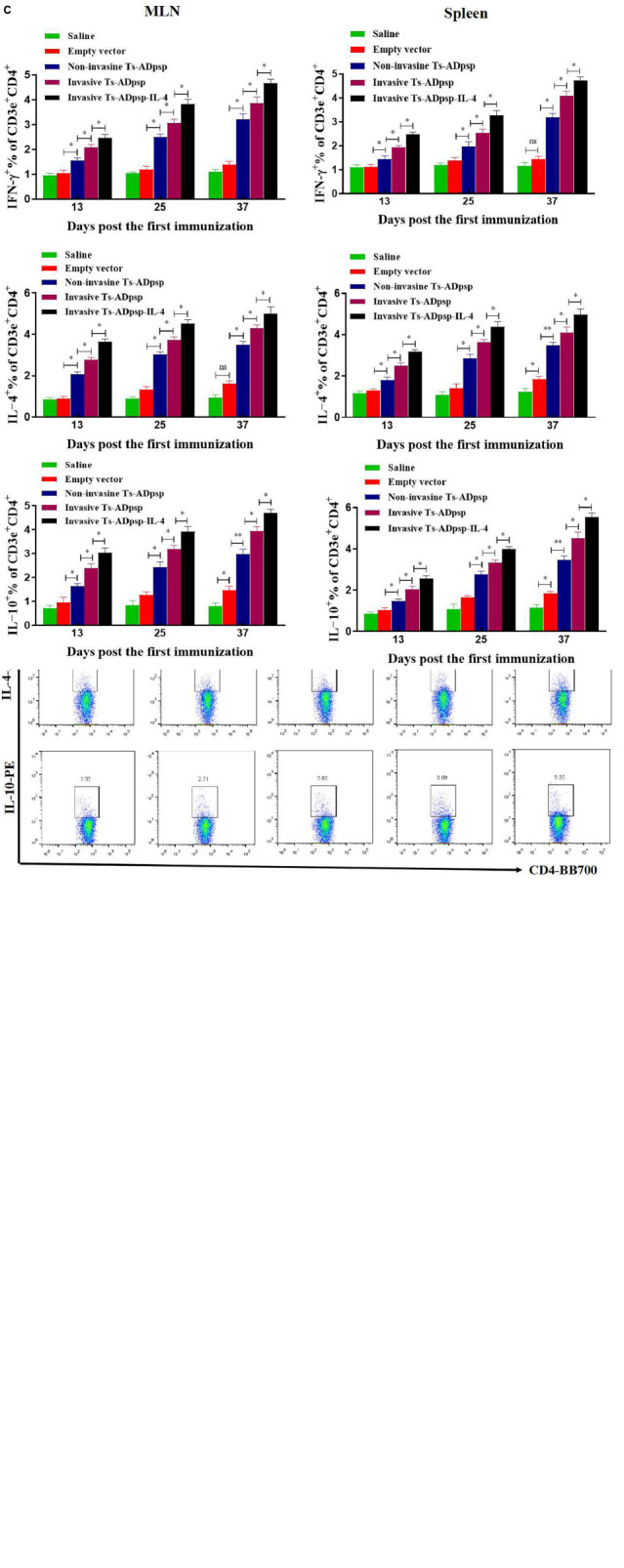
Cytokine secretion following vaccination. IFN-γ, IL-4, and IL-10 levels in MLN **(A)** and spleen **(B)** of vaccinated mice were evaluated by flow cytometry 10 days after each immunization. **(C)** Quantification of IFN-γ, IL-4, and IL-10 cytokines. Data are presented as mean ± SEM (*n* = 5 mice per group). *P < 0.05, **P < 0.01.

The levels of the cytokines IFN-γ, IL-4, and IL-10 in mesenteric lymph node (MLN) cells and splenocytes were evaluated by flow cytometry 10 days after each immunization (Figure 6). The secretion of IFN-γ, IL-4, and IL-10 was increased in both cell types stimulated with Ts-ADpsp and Ts-ADpsp-IL-4 compared with the control groups (*P* < 0.05, < 0.01, and < 0.001), indicating that a Th1/Th2 mixed immune response was induced by the pValac-Ts-ADpsp/pSIP409, pValac-Ts-ADpsp/pSIP409-FnBPA, and pValac-Ts-ADpsp-IL-4/pSIP409-FnBPA DNA vaccines. Moreover, IFN-γ (*P* < 0.05, < 0.01, and < 0.001), IL-4 (*P* < 0.05, < 0.01, and < 0.001), and IL-10 (*P* < 0.05, *P* < 0.01 and < 0.001) levels were highest in the invasive *L. plantarum* Ts-ADpsp-IL-4 group. These results were consistent with the findings from ELISA.

### Assessment of the Protective Effect of the DNA Vaccine

**FIGURE 7:**
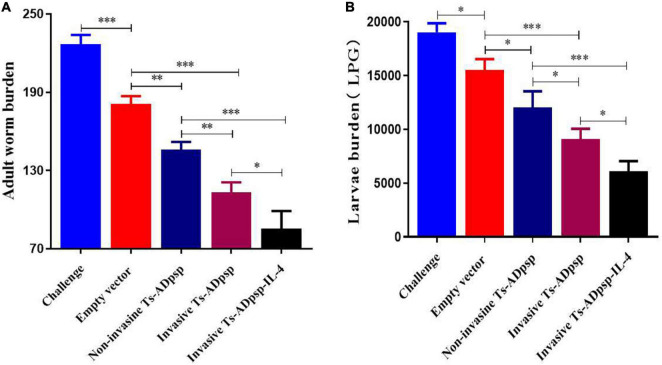
Worm burden in mice following *T. spiralis* infection with or without vaccination. Adult worm **(A)** and larvae per gram of muscle (LPG) burden **(B)** of vaccinated mice after infection with 350 *T. spiralis* muscle larvae. Data are presented as mean ± SEM (*n* = 8 mice per group). *P < 0.05, **P < 0.01, and ***P < 0.001.

To evaluate the protective effect of recombinant *L. plantarum*, we examined adult worm and muscle larvae burdens in immunized mice at 6 days post-infection (dpi) and 35 dpi separately. Adult worm burden was reduced by 20.3% (pValac/pSIP409), 35.7% (pValac-Ts-ADpsp/pSIP409), 50.1%(pValac-Ts-ADpsp/pSIP409-FnBPA), and 62.4% (pValac-Ts-ADpsp-IL-4/pSIP409-FnBPA) compared with infected but non-immunized mice (all *P* < 0.05; Figure 7A). Similarly, muscle larvae burden was decreased by 13.6% (pValac/pSIP409), 36.9% (pValac-Ts-ADpsp/pSIP409), 49.2% (pValac-Ts-ADpsp/pSIP409-FnBPA), and 60.5% (pValac-Ts-ADpsp-IL-4/pSIP409-FnBPA) compared with the negative control (all *p* < 0.05; Figure 7B). These results indicated that invasive *L. plantarum* expressing Ts-ADpsp-IL-4 conferred greater protection against *T. spiralis* infection than non-invasive *L. plantarum*.

### Histopathologic Analysis

**FIGURE 8:**
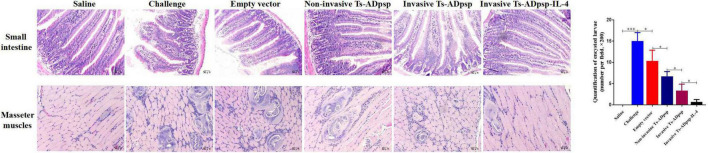
Histopathologic analysis of small intestine and masseter muscles after *T. spiralis* infection. Mice were challenged with 350 *T. spiralis* muscle larvae, and histologic analysis was performed by hematoxylin and eosin staining (200 × magnification). Quantification of encysted larvae. *P < 0.05, **P < 0.01, and ***P < 0.001.

Small intestine specimens of mice euthanized at 6 dpi and masseter muscle specimens of mice euthanized at 35 dpi were embedded in paraffin and sectioned to evaluate histopathologic changes. A larger number of goblet cells, shorter and thicker intestinal villi, and eosinophilic infiltration were observed in the small intestine of mice infected with *T. spiralis* muscle larvae only (Figure 8). However, the villus structure of mice in the invasive and non-invasive *L. plantarum* Ts-ADpsp and invasive *L. plantarum* Ts-ADpsp-IL4 groups showed less damage with a smaller number of infiltrating goblet cells and eosinophils, especially in the invasive *L. plantarum* Ts-ADpsp-IL4 group. Furthermore, there were encysted larvae in the masseter muscles of mice in all groups except for the saline control group, resulting in varying degrees of destruction of muscle fibers. Meanwhile, the muscle fibers of the invasive *L. plantarum* Ts-ADpsp-IL-4 group were intact, with less encysted larvae than in the invasive and non-invasive *L. plantarum* Ts-ADpsp groups.

## Discussion

As a multicellular parasitic nematode, *T. spiralis* has numerous antigens. To avoid the host’s immune surveillance system and achieve successful colonization, *T. spiralis* uses a strategy of constantly changing antigens (Bruschi and Chiumiento, 2012). This prevents the development of immunity against *T. spiralis* and makes the diagnosis of trichinellosis challenging. In nucleic acid vaccines, an exogenous gene encoding an antigenic determinant is placed under the control of eukaryotic expression elements and the recombinant plasmid is introduced into a host, which then mounts a specific immune response to the antigen (Zhang et al., 2018). Different proteases secreted by parasites have various functions such as tissue penetration, larval migration, inhibition of blood clotting, digestion, molting, and extracellular matrix degradation; thus, epitopes for nucleic acid vaccines can be designed based on protease (Boulard, 1989; Anisuzzaman et al., 2012).

In this study, the adult worm-specific hypothetical serine protease (Ts-ADpsp) of *T. spiralis* was selected as the antigen for vaccine development. *L. plantarum* carrying the recombinant plasmid entered the intestinal epithelial cells of mice and induced an immune response. The pValac-Ts-ADpsp eukaryotic expression system ensured that the structure and biological activity of the recombinant and native proteins were similar. The expression of Ts-ADpsp protein from pValac-Ts-ADpsp delivered by *L. plantarum* was previously shown to be superior to the *E. coli* TspGST prokaryotic expression system in terms of stimulating IgG production, cellular immunity, and worm burden (Liu et al., 2017). Although vaccination with TsPmy DNA delivered by live attenuated *Salmonella typhimurium* conferred protection against *T. spiralis* infection, *L. plantarum* may be a safer vaccine carrier (Wang et al., 2016). Our previous study has reported that FnBPA could be successfully expressed in *L. plantarum* NC8 (Liu et al., 2019). Interestingly, invasive *L. plantarum* has a higher invasion rate than the non-invasive strain because it expresses FnBPA, which binds α5β1 integrin on the host cell surface (Pontes et al., 2012; Liu et al., 2019). Thus, FnBPA improves the efficiency of recombinant plasmid delivery by *L. plantarum* as well as antigen presentation and vaccine efficacy. In addition, the DNA vaccines in this study are safe and stable for the mouse. On one hand, LAB, which belongs to food-grade bacteria, is considered to be probiotic strains in most cases (Diep et al., 2009), with potential benefits to human and animal health. On the other hand, DNA vaccines could themselves maintain constancy in the plasmid components or backbone because they are not produced from vector immunity in the host and they do not integrate into the host genome (Ghaffarifar, 2018).

IgG plays an important role in protective immunity against *T. spiralis* infection. Intramuscular injection of *T. spiralis* newborn larva (NBL) strongly stimulated a specific IgG response in mice and reduced muscle larvae burden by 77.93% (Xu et al., 2017). IgG titer was elevated in mice vaccinated with Ts-Adsp, which enhanced protection against *T. spiralis* (Xu et al., 2020). In this study, oral vaccination of mice with recombinant *L. plantarum* carrying Ts-ADpsp antigen increased anti–Ts-ADpsp–specific IgG titer after each immunization, with a peak after the final immunization; the titer was higher in mice vaccinated with invasive *L. plantarum* expressing FnBPA compared to non-invasive *L. plantarum* Ts-ADpsp.

The intestinal mucosa is the first barrier against intestinal pathogens such as *T. spiralis*. sIgA against *T. spiralis* adult worm surface proteins was shown to mediate adult worm expulsion from the intestine (Wang et al., 2017). In the present study, oral administration of pValac-Ts-ADpsp/pSIP409, pValac-Ts-ADpsp/pSIP409-FnBPA, and pValac-Ts-ADpsp–IL-4/pSIP409-FnBPA strongly induced specific IgA production in the intestinal mucosa of mice, with the maximum titer measured after the third boost immunization. LAB can enter sites of induction of the mucosal immune response such as Peyer’s patches to stimulate sIgA production (Wang et al., 2014; Liu et al., 2020). We found that the empty vector induced specific sIgA secretion in mice compared to saline, suggesting that *L. plantarum* itself contributes to the activation of an immune response.

IFN-γ induces eosinophil activation and enhances the cytotoxicity of macrophages against *T. spiralis* NBL, while IL-4 participates in the excretion of *T. spiralis* adult worm. Thus, a mixed Th1/Th2 response mediates protection against *T. spiralis* infection (Miahipour et al., 2016; Tharmalingam et al., 2016). We measured Th1 (IFN-γ) and Th2 (IL-4 and IL-10) cytokine levels in the spleen and MLNs of vaccinated mice 10 days after each immunization by indirect ELISA and flow cytometry, respectively, and the results showed that Th1 and Th2 cytokine levels were elevated and reached a peak 10 days after the last immunization, indicating that the vaccine stimulated both Th1 and Th2 cellular immune responses, especially in the invasive *L. plantarum* Ts-ADpsp-IL-4 group. IL-4 is an important regulator of cellular and humoral immunity and can confer protection against *T. spiralis* infection (Wang et al., 2020); accordingly, we observed that immunization with pValac-Ts-ADpsp-IL-4/pSIP409-FnBPA significantly reduced intestinal damage and worm burden compared to pValac-Ts-ADpsp/pSIP409-FnBPA administration.

The present study exhibited that oral vaccination with Ts-ADpsp DNA vaccine displayed 62.4% adult worm reduction and 60.5% muscle larvae reduction after *T. spiralis* challenge relative to the saline control group, suggesting Ts-ADpsp gene stimulated specific immunity and provided a partial protection against *T. spiralis* infection. However, in a previous report when a recombinant serine protease from adult worms was used in combination with an alum adjuvant to immunize mice, which were subsequently challenged with *T. spiralis* larvae, the animals exhibited an average reduction in the muscle larvae burden of 46.5% relative to the control group (Feng et al., 2013). The reduction in muscle larvae burden in this work was lower than above report, which may be due to invasive *L. plantarum* expressing FnBPA binding to the α5β1 integrin on the surface of host cells to deliver DNA vaccines directly or across an epithelial cell monolayer into DCs, thereby enhancing the ability of antigen presentation and efficacy of vaccine protection (Long et al., 2014). The DNA vaccine in the present study, with a lower protective effect compared with previous studies, provides a promising strategy for the development of an oral DNA vaccine against *T. spiralis* infection. *T. spiralis* is a unique intracellular parasitic nematode with a complicated life cycle, and the antigens against the host were stage specific. Vaccination with a single antigenic molecule produced a partial protection in the host, which might not be enough to resist *T. spiralis* infection (Liu et al., 2015; Ortega-Pierres et al., 2015). Hence, the novel immune strategy, vaccination routes, and multivalent antigenic molecules from different life stages should be explored in the future to enhance the protective efficacy of *T. spiralis* vaccine.

Invasive *L. plantarum* expressing FnBPA and IL-4 strain could effectively colonize and act as adhesive host cavities, ensuring long-lasting persistence in the host and possibly extending the time of antigen presentation to the immune system to induce anti-ADpsp specific systemic and mucosal immune responses against *T. spiralis* infection. Furthermore, IL-4 could induce T cell and B cell differentiation and proliferation, accelerate smooth muscle contraction, and promote the elimination of intestinal *T. spiralis*.

## Conclusion

In summary, the results of our study demonstrate that oral vaccination with pValac-Ts-ADpsp/pSIP409 delivered by invasive *L. plantarum* induced anti-Ts-ADpsp-specific IgG production and stimulated the mucosal and cellular immune responses in mice, thereby alleviating intestinal damage and reducing worm burden following infection with *T. spiralis*. The protective effect was enhanced by IL-4 used as an adjuvant. These findings provide evidence for the efficacy of a novel DNA vaccine for trichinellosis.

## Materials and Methods

### Parasites and Experimental Animals

The *T. spiralis* isolate ISS534 was maintained by serial passaging in ICR mice in the laboratory of animal care in the center of Jilin Agricultural University (Jilin, China). Individual *T. spiralis* larvae were isolated from mice infected for more than 35 dpi by digesting mouse carcasses with 1% HCl and 1% pepsin (1:3,000; Solarbio, Beijing, China; Li et al., 2010). Experimental mice were orally administered 350 muscle larvae. The 8-week-old male BALB/c mice were obtained from Hua Fu Kang Biotechnology (Beijing, China) and allowed to eat and drink freely until the end of the experiment. The use of mice in this study was approved by the Animal Ethics Committee and Institutional Life Science Ethics Committee of Jilin Agriculture University (permit no. JLAU 20200904001). The experiments were carried out in strict accordance with the National Laboratory Animal Welfare Guidelines. If any animal has labored breathing, bleeding diarrhea, or becomes moribund it will be euthanized immediately by CO_2_ inhalation.

### Bacterial Strains and Cells Culture

*Escherichia coli* TG1 cells (Takara Bio, Otsu, Japan) were cultured in LB broth at 37°C for 24 h. Invasive and non-invasive *L. plantarum* NC8 were generously provided by Jing Liu (Jilin Agricultural University, Changchun, China) and grown in de Man, Rogosa, and Sharpe (MRS) medium with erythromycin (10 μg/ml) at 30°C under anaerobic conditions. Invasive *L. plantarum* NC8 strain expressing protein FnBPA could enhance the adhesion and invasion ratios of *L. plantarum* strain on a porcine intestinal epithelial cell line (IPEC-J2) compared with non-invasive *L. plantarum* NC8 (Liu et al., 2019). HEK-293T cells were cultured in complete Dulbecco’s Modified Eagle’s Medium (Gibco, Grand Island, NY, United States) at 37°C and 5% CO_2_.

### PCR Amplification of Ts-ADpsp and Ts-ADpsp-IL-4

The DNA sequences of Ts-ADpsp (GenBank: EU263332.1) and murine IL-4 (mIL-4) (GenBank: AAR87867.1) were obtained from GenBank. The mIL-4 gene was synthesized by Sangon Biotech (Shanghai, China) and cloned into the pUC57 vector. Total RNA was extracted using TRIzol reagent (Invitrogen, Carlsbad, CA, United States) from adult worms of *T. spiralis*. Specific primer (forward, 5′-GCGAATTCTGCAGGATCCAACTTTTTTTTTTTTTTTTTT-3′) was used to reverse transcribe the RNA into cDNA, which was used as a template for PCR. Ts-ADpsp and mIL-4 genes were amplified using Ts-ADpsp primers (forward, 5′-GGGGTACCATGAAGAGGTTTCACCACCACCACTTCGG-3′; reverse, 5′-GCTCTAGAGTACACGGCGCCGCTGAT-3′) and IL-4 primers (forward, 5′-ATGGGCGTGAAGGTGCTGTT-3′; reverse, 5′-GTGATGGTGATGGTGGTGG GA-3′) and fused with Flag and His tags, respectively, with *Kpn*I and *Xba*I sites added to the 5′ and 3′ ends, respectively. Ts-ADpsp and mIL-4 were joined together *via* the P2A linker sequence by PCR using Ts-ADpsp-IL-4 primers (forward, 5′-GGGGTACCACCGGTCGCCACCATGG-3′; reverse, 5′-GCTCTAGATTAATGG TGGTGATGGTGGTGGCTCA-3′) to produce the Ts-ADpsp-IL-4 fragment.

### Recombinant Plasmids Construction

The eukaryotic expression vector pValac-green fluorescent protein (GFP; Institut National de la Recherche Agronomique, Paris, France) was digested with *Kpn*I and *Xba*I to remove the GFP gene and purified using the DNA Fragment Purification Kit (Axygen, Taizhou, China). The Ts-ADpsp and Ts-ADpsp-IL-4 fragments were separately linked with the purified product to obtain the recombinant plasmids pValac-Ts-ADpsp and pValac-Ts-ADpsp-IL-4. These along with the empty vector (pValac-GFP) were transformed into *E. coli* TG1 and cultured on LB agar with 10 μg/ml ampicillin to select positive clones, which were verified by PCR amplification and restriction enzyme digestion.

### Detection of Recombinant Protein Expression by Immunofluorescence Analysis and Western Blotting

For immunofluorescence analysis, HEK-293T cells were cultured in a 24-well cell plate containing glass coverslips treated with 1% polylysine at 37°C with 5% CO_2_ until the cells reached 80% confluence. The cells were transfected with pValac-GFP, pValac-Ts-ADpsp, or pValac-Ts-ADpsp-IL-4 plasmid using Nano293T transfection reagent (NCM Biotech, Newport, RI, United States). After 48 h, the cells were washed with phosphate-buffered saline (PBS), fixed with 4% paraformaldehyde, incubated with 0.1% Triton X-100, and blocked with 5% skimmed milk. The cells were then incubated with anti-Flag (1:2,000 dilution) or anti-His (1:2,000 dilution) monoclonal antibody (Proteintech Group, Inc., Rosemont, IL, United States) at room temperature for 1 h, except for the pValac-GFP group (positive control). After washing with PBS containing 0.1% Triton X-100 (PBST) and incubating with fluorescein isothiocyanate-labeled goat anti-mouse IgG (1:1,000 dilution) at room temperature for 1 h, the cells were stained with 4′,6-diamidino-2-phenylindole (1:3,000 dilution) and fluorescence was observed by confocal microscopy (Leica, Wetzlar, Germany).

For western blotting, HEK-293T cells were cultured in a 48-well plate for 12 h before transfection; at 48 h after transfection, the cells were lysed with radioimmunoprecipitation assay lysis buffer (Beyotime) at 4°C for 20 min and centrifuged. The target protein in the supernatant was separated by 10% SDS-PAGE and transferred to a polyvinylidene difluoride (PVDF) membrane, which was blocked with 3% bovine serum albumin (BSA) at room temperature for 2 h and then probed with anti-Flag (1:1,000 dilution) and anti-His (1:1,000 dilution) antibodies followed by horseradish peroxidase (HRP)-conjugated goat anti-mouse IgG (Proteintech Group, Inc., Rosemont, IL, United States). Protein bands were visualized and imaged using an Amersham Imager 600 RGB (GE Healthcare Life Sciences, Marlborough, MA, United States).

### Plasmid DNA Vaccine Construction

Invasive or non-invasive *L. plantarum* NC8 competent cells were transformed with the recombinant plasmids pValac-GFP, pValac-Ts-ADpsp, and pValac-Ts-ADpsp-IL4 using a Gene Pulser electroporator (Bio-Rad, Hercules, CA, United States). The cells were grown on MRS agar supplemented with chloramphenicol (Cm; 10 μg/ml) and erythromycin (Em; 10 μg/ml) to select transformants, which were confirmed by PCR amplification and sequencing. Clones with the correct insertions were named pValac/pSIP409, pValac-Ts-ADpsp/pSIP409, pValac-Ts-ADpsp-IL-4/pSIP409, pValac-Ts-ADpsp/pSIP409-FnBPA, and pValac-Ts-ADpsp-IL-4/pSIP409-FnBPA and cultured in MRS medium containing Cm and Em (Cm + Em) without shaking at 30°C for 3 h until the optical density at 600 nm (OD_600_) was 0.2–0.3. The expression of proteins ADpsp and IL-4 were induced with sakacin P (SppIP), followed by a continuous culture at 30°C in an anaerobic chamber for 36 h (OD_600_ = 1). Recombinant *L. plantarum* was harvested and centrifuged at 8,000 × *g* for 10 min at 4°C and the liquid medium was discarded. The bacterial pellet was then washed twice using sterile saline and the bacterial pellet was resuspended in saline with a final cell density of 5 × 10^9^ CFU/ml. A total of 200 μL of the bacterial suspension (1 × 10^9^ CFU) was used to orally immunize the experimental animal.

### Measurement of Recombinant *Lactiplantibacillus plantarum* Invasion Rate

Recombinant *L. plantarum* (pValac-Ts-ADpsp/pSIP409, pValac-Ts-ADpsp/pSIP409-FnBPA, pValac-Ts-ADpsp–IL-4/pSIP409, and pValac-Ts-ADpsp–IL-4/pSIP409-FnBPA) cells were cocultured with baby hamster kidney (BHK)-21 cells in a 24-well plate for 2 h until the multiplicity of infection was 10^3^ bacteria/cell. The extracellular bacteria were inactivated with gentamicin (20 mg/ml). The cells were then washed using PBS and cultured on solid MRS (Cm + Em) for 24 h and the number of colonies was counted.

### Immunization Schedule and Challenge

**FIGURE 9:**
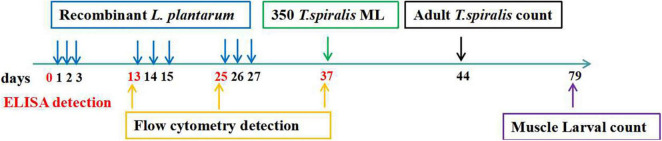
Immune procedures. The immune-stimulation was carried out three times (1, 2, 3; 13, 14, 15; 25, 26, 27) at a 10-day interval and orally administered with 350 *T. spiralis* muscle larvae at day 37 after the first immunization. The ELISA detection was performed at days 0, 13, 25, 37 after the first immunization to detect the humoral immunity and mucosal immune response induced by recombined *L. plantarum* pValac-Ts-ADpsp-IL-4/pSIP409-FnBPA. The flow cytometry was carried out at days 13, 25, 37 after the first immunization to assess the cellular immune response. Adult worm was counted at days 44 after the first immunization, and the muscle larvae was performed at day 79 after the first immunization to assess the protective effect of recombinant DNA vaccine against *T. spiralis* infected.

BALB/c mice were vaccinated 3 times at 10-day intervals. The immunization schedule and challenge doses are presented in Table 1. Briefly, experimental mice were randomly divided into the following 5 groups: (1) non-invasive *L. plantarum* Ts-ADpsp (pValac-Ts-ADpsp/pSIP409); (2) invasive *L. plantarum* Ts-ADpsp (pValac-Ts-ADpsp/pSIP409-FnBPA); (3) invasive *L. plantarum* Ts-ADpsp-IL-4 (pValac-Ts-ADpsp-IL-4/pSIP409-FnBPA); (4) empty vector *L. plantarum* (pValac/pSIP409); and (5) saline control. The experimental groups were orally vaccinated with 10^9^ CFU recombinant *L. plantarum* (pValac-Ts-ADpsp/pSIP409, pValac-Ts-ADpsp/pSIP409-FnBPA, pValac-Ts-ADpsp-IL-4/pSIP409-FnBPA, or pValac/pSIP409) by oral gavage and the control mice were given saline (Wang et al., 2020). To evaluate the humoral and cellular immune responses, blood, intestinal lavage, spleen, and mesenteric lymph node (MLN) samples of mice (*n* = 5) of each group were obtained from mice 10 days after each immunization. To evaluate the protective effect of recombinant *L. plantarum* against *T. spiralis* infection, the mice were orally inoculated with 350 individual *T. spiralis* larvae 10 days after the last immunization. The adult worms were collected from the intestines of infected mice (*n* = 8) at 6 day following *T. spiralis* challenge. The muscle larvae were harvested from the muscle tissue of infected mice (*n* = 8) at 35 dpi. The flow chart of immune procedures is exhibited in Figure 9.

**TABLE 1:** Immunization schedule and challenge doses.

Groups	Recombinant *L. plantarum* strains	Number	Immunization (dose)	Challenge (dose)
Saline	/	36	Normal saline/200 μL	Unchallenged
Challenge	/	36	Normal saline/200 μL	350 *T. spiralis* muscle larvae
Empty vector	pValac/PSIP409	36	1.0 × 10^9^ CFU/200 μL	350 *T. spiralis* muscle larvae
Non-invasive Ts-ADpsp	pValac-Ts-ADpsp/pSIP409	36	1.0 × 10^9^ CFU/200 μL	350 *T. spiralis* muscle larvae
Invasive Ts-ADpsp	pValac-Ts-ADpsp/pSIP409-FnBPA	36	1.0 × 10^9^ CFU/200 μL	350 *T. spiralis* muscle larvae
Invasive Ts-ADpsp-IL-4	pValac-Ts-ADpsp-IL4/pSIP409-FnBPA	36	1.0 × 10^9^ CFU/200 μL	350 *T. spiralis* muscle larvae

### Antigen Preparation

The purified PCR products of Ts-ADpsp were cloned into the pUC57 vector and sub-cloned into the expression vector pET-30a. The transformants were cultured on LB agar with kanamycin (Kan; 50 μg/ml) to select positive clones, which were verified by enzymatic digestion and confirmed by DNA sequencing. The expression of the Ts-ADpsp protein was induced with 0.5 mM IPTG for 16 h at 28°C. The recombinant Ts-ADpsp protein was expressed in the supernatant and purified by HisPurTM Ni-NTA Spin Columns. The protein concentration of purified Ts-ADpsp protein was determined by Bradford method and analyzed by western blotting using a 5% acrylamide stacking gel and 12% acrylamide separating gel.

### Detection of Mucosal and Systemic Antibody Responses

At 10 days after each immunization, a blood sample was collected from mice and the serum was separated and stored at −80°C. Intestinal lavage was performed as previously described (Wang et al., 2006). Briefly, mice were sacrificed and the abdomen was cut open aseptically; approximately 15 cm of the small intestine was cut longitudinally and washed twice with cold PBS and then centrifuged for 10 min at 3,000 × *g*. The supernatant was stored at −80°C.

Specific IgG and sIgA were detected by enzyme-linked immunosorbent assay (ELISA) using 96-well plates that were coated with purified Ts-ADpsp antigen (5 μg/ml) at 4°C for 12 h, washed 4 times with PBST, and blocked with 3% BSA at 37°C for 2 h. Serum and intestinal lavage served as the source of primary antibody and HRP-conjugated goat anti-mouse IgG/sIgA (CWBio, Beijing, China) was used as the secondary antibody. Total intestinal sIgA, interferon (IFN)-γ, IL-4, and IL-10 levels were detected with a commercial ELISA kit (Jiangsu Meimian Industrial Co., Zhangjiagang, China).

### Cytokine Detection by Flow Cytometry

Spleen and MLN samples isolated from mice 10 days after each immunization were used to assess cellular immune responses. Dissociated spleen and MLN cells stimulated with Ts-ADpsp protein (5 μg/ml) for 8 h were added to GolgiPlug™ (Brefeldin A) for an additional 4 h at 37°C with CO_2._ Then, the cells were collected and incubated with Alexa 700-conjugated monoclonal rat anti-mouse cluster of differentiation (CD)3e and BB700-conjugated monoclonal rat anti-mouse CD4 antibodies for 45 min at 4°C. The cells were fixed, permeabilized, and stained with phycoerythrin (PE)-Cy7-conjugated rat anti-mouse IFN-γ, allophycocyanin-conjugated rat anti-mouse IL-4, and PE-conjugated rat anti-mouse IL-10 monoclonal antibodies. Finally, the samples were detected by flow cytometry (BD Biosciences, Franklin Lakes, NJ, United States).

### Adult Worm and Muscle Larvae Isolation and Worm Burden Evaluation

The abdomen of the euthanized mice were cut aseptically and the small intestines were collected to isolate adult worm at 6 dpi with *T. spiralis.* The small intestine was cut longitudinally, washed using pre-warmed saline, cut into pieces, and incubated in saline at 37°C for 2 h. Then, the released adult worms were separated from the intestinal debris by filtration through a 200-mesh sieve and differential sedimentation for 30 min. Adult worms were centrifuged and the worms in the sediment were collected. In addition, the muscle larvae were isolated from the muscle tissue according to the standard artificial digestion method (Li et al., 2010). Briefly, mice were euthanized and dissected to remove visceral tissues and the muscle tissues were collected at 35 dpi. The muscle tissues were cut into small pieces and digested using 1% HCl and 1% pepsin (Solarbio, Beijing, China) at 37°C for 2 h under continuous agitation using an electric stirrer and the digested mixture was passed through a 50-mesh sieve to remove the coarse particles. Muscle larvae were collected on a 200-mesh sieve, washed twice using saline, and then suspended in saline. The supernatant fluid was discarded and the muscle larvae in sediment were counted under the microscope (Leica, Wetzlar, Germany).

The reduction evaluation in adult worm and muscle larvae was based on the mean number of adult worm or muscle larvae collected from the group vaccinated with empty vector, non-invasive Ts-ADpsp, invasive Ts-Adpsp, invasive Ts-Adpsp-IL-4, or both compared with those from the saline control group using the following formula: % adult worm reduction = (1-mean number of adult worm in vaccinated mice/mean number of adult worm in control mice) × 100%, % Larva reduction = (1-mean number of larvae per gram muscle in vaccinated mice/mean number of larvae per gram muscle in control mice) × 100%.

### Statistical Analyses

Data were analyzed with Prism 8 software (GraphPad, La Jolla, CA, United States) and are expressed as mean ± standard deviation of at least 3 independent experiments. Differences between groups were evaluated by one-way analysis of variance. *P* < 0.05 was considered statistically significant.

## Data Availability Statement

The original contributions presented in the study are included in the article/supplementary material, further inquiries can be directed to the corresponding author/s.

## Author Contributions

YX and BZ carried out the literature search and drafted the first version of the manuscript. NW, H-BH, YQ, and H-NL were responsible for designing and analyzed the data. Z-YZ, J-YL, T-XP, YT, Y-LJ, and C-WS performed the experiments and assisted on the sample collections. G-LY and C-FW revised the review. All authors read and approved the final manuscript.

## Conflict of Interest

The authors declare that the research was conducted in the absence of any commercial or financial relationships that could be construed as a potential conflict of interest.

## Publisher’s Note

All claims expressed in this article are solely those of the authors and do not necessarily represent those of their affiliated organizations, or those of the publisher, the editors and the reviewers. Any product that may be evaluated in this article, or claim that may be made by its manufacturer, is not guaranteed or endorsed by the publisher.
